# Control of Surface Segregation in Bimetallic NiCr Nanoalloys Immersed in Ag Matrix

**DOI:** 10.1038/srep19153

**Published:** 2016-01-11

**Authors:** Murtaza Bohra, Vidyadhar Singh, Panagiotis Grammatikopoulos, Evropi Toulkeridou, Rosa E. Diaz, Jean-François Bobo, Mukhles Sowwan

**Affiliations:** 1 Nanoparticles by Design Unit, Okinawa Institute of Science and Technology Graduate University, 1919-1 Tancha Onna-Son, Okinawa, 904-0495, Japan; 2 Mahindra Ecole Centrale, Survey no: 62/1A, Bahadurpally Jeedimetla, Hyderabad-500043, Telangana India; 3 Centre d’Elaboration de Materiaux et d’Etudes Structurales (CEMES), 29 rue Jeanne Marvig, 31055 Toulouse Cedex 4, France; 4 Nanotechnology Research Laboratory, Al-Quds University, East Jerusalem, P.O. Box 51000, Palestine

## Abstract

Cr-surface segregation is a main roadblock encumbering many magneto-biomedical applications of bimetallic M-Cr nanoalloys (where M = Fe, Co and Ni). To overcome this problem, we developed Ni_95_Cr_5_:Ag nanocomposite as a model system, consisting of non-interacting Ni_95_Cr_5_ nanoalloys (5 ± 1 nm) immersed in non-magnetic Ag matrix by controlled simultaneous co-sputtering of Ni_95_Cr_5_ and Ag. We employed Curie temperature (*T*_*C*_) as an indicator of phase purity check of these nanocomposites, which is estimated to be around the bulk Ni_95_Cr_5_ value of 320 K. This confirms prevention of Cr-segregation and also entails effective control of surface oxidation. Compared to Cr-segregated Ni_95_Cr_5_ nanoalloy films and nanoclusters, we did not observe any unwanted magnetic effects such as presence Cr-antiferromagnetic transition, large non-saturation, exchange bias behavior (if any) or uncompensated higher *T*_*C*_ values. These nanocomposites films also lose their unique magnetic properties only at elevated temperatures beyond application requirements (≥800 K), either by showing Ni-type behavior or by a complete conversion into Ni/Cr-oxides in vacuum and air environment, respectively.

One of the foremost driving forces of current nano/biotechnology research is the ever-increasing need for new and smart magnetic nanomaterials that can be employed in a variety of applications encompassing magnetic resonance imaging (MRI), targeted drug delivery, giant magneto-resistive (GMR) sensors, induction-heating self-temperature controlling systems, etc.^1^,^2^,^3^. Often, a first line of attack in designing nanomaterials with tailored properties is to screen bulk material attributes for inspiration. Thus, the Ni_95_Cr_5_ alloys^1^, showing a low Curie temperature (*T*_*C*_ = ~320 K), are certainly a very attractive candidate for several of the aforementioned applications.

Once a promising alloy has been selected, a nanostructure has to be designed and fabricated that maintains the desirable physical and chemical properties of the bulk reference system. However, synthesis of the Ni_95_Cr_5_ nanoalloy with the desired *T*_*C*_ is rather challenging to start with, owing to a strong tendency for elemental demixing. Various types of inhomogeneous structures thus emerge, exhibiting *T*_*C*_ higher than the bulk value, and, in some cases, even attaining a pure-Ni bulk *T*_*C*_ value (~ 625 K), depending upon growth conditions^4^,^5^. For example, a recent study by the authors demonstrated the detrimental effect of element-specific Cr-surface segregation in vacuum; both NiCr alloy nanoparticles and NiCr thin films grown by gas-phase synthesis methods yielded high Ni-rich segregates of prohibitively high *T*_*C*_ values (>470−500 K)^6^. The origin of Cr-segregation was theoretically explained mainly on the basis of favorable energetics, since it resulted in overall potential energy minimization^6^. Ban *et al.* recently reported on successfully synthesizing by mechanical milling NiCr nanoalloys that show a low *T*_*C*_  ~ 325 K, even though the increased Cr concentration they reported (Ni_75_Cr_25_) corresponds to a bulk alloy that displays non-magnetic behavior (≥13 *at*.% Cr)^4^. They attributed this unexpected behavior to extensive heterogeneity in particle size distribution and composition, which is inherent to the fabrication method. Unfortunately, though, the *T*_*C*_ of their nanoparticles increased significantly when the samples were applied in a hyperthermia experiment, exactly due to this extensive heterogeneity.

Consequently, stability under working conditions is the ultimate criterion NiCr nanoalloys have to fulfill; otherwise, the end-product is merely an academic exercise. The nanostructure has to be tested with respect to its stability under realistic operational conditions, to assess its applicability range and determine its limitations. For example, air exposure is a common source of degradation for the magnetic properties of nanoalloys, as it induces selective oxidation and facilitates further segregation. When NiCr nanoalloys are exposed to air at ambient temperature, oxidation behavior is complicated and influenced by both Ni and Cr oxidation energies and rates of diffusion; in particular, by high preferential oxidation of Cr ions due to the high mobility of Cr in the host Ni matrix^5^,^7^. When the concentration of Cr is high, a Cr_2_O_3_ surface layer forms, that potentially have some merits (e.g. for high corrosion-resistance applications)^3^,^8^. At 5% Cr, however, the full protective oxide layer cannot form; multi-site nucleation and coalescence of oxide particles ensues, leading to a core-satellite structure with possible cavity formation within the nanoparticle due to Kirkendall effect^7^, and ultimately resulting in deterioration of magnetic properties^9^. Annealing can also act as an additional degradation agent, enhancing demixing and converting Ni_95_Cr_5_ nanoalloys into core-shell or -satellite type structures, instead of restoring the expected bulk magnetic structure^6^.

Therefore, precise control of elemental segregation is the key to maintain a desirable magnetic behavior. Various methods have been proposed to protect the magnetic nanoparticles/nanoalloys from surface oxidation^10^,^11^,^12^,^13^,^14^. Capping layers of metals are, generally, assumed to be a good barrier against oxidation, but recent findings by the authors^11^ and work by Koch *et al.*^12^ showed that post-deposition capping by noble metal Ag (~80 nm) is insufficient to shield Co nanoparticles (~7−14 nm in diameter) from surface oxidation, with the resultant effects in their magnetic properties. In contrast, De Toro *et al.*^15^ demonstrated that diluted (<10 *at.*%) cluster-assembled granular Co:Cu films, prepared by simultaneous co-deposition of Co clusters with a Cu vapor, are perfectly stable under ambient conditions. Inspired by this work, and taking into account biocompatibility requirements for Ni_95_Cr_5_ applications, herein we report on the development via simultaneous co-sputtering of Ni_95_Cr_5_:Ag nanocomposites with bulk Curie temperature values (*T*_*C*_ = 320 K) and full control of Cr-segregation under working conditions.

## Results

In the present study, we synthesized Ni_95_Cr_5_:Ag nanocomposites using a co-sputtering process. As estimated by energy dispersive x-ray spectroscopy obtained with the Titan transmission electron microscope (TEM), they exhibit relative atomic concentrations around Ni_95_Cr_5_ (~35%): Ag (~65%) (shown in supplementary Fig. S1a). Due to the abundance of Ag in our sample, as depicted in the high-resolution TEM (HRTEM) image (Fig. S1b), direct observation of the NiCr clusters alone is difficult. However, the strong correlation between structural and magnetic properties enables one to gather valuable information about the presence and structure of Ni_95_Cr_5_ nanoalloy clusters embedded into the Ag matrix by conducting magnetic measurements.

**Figure 1:**
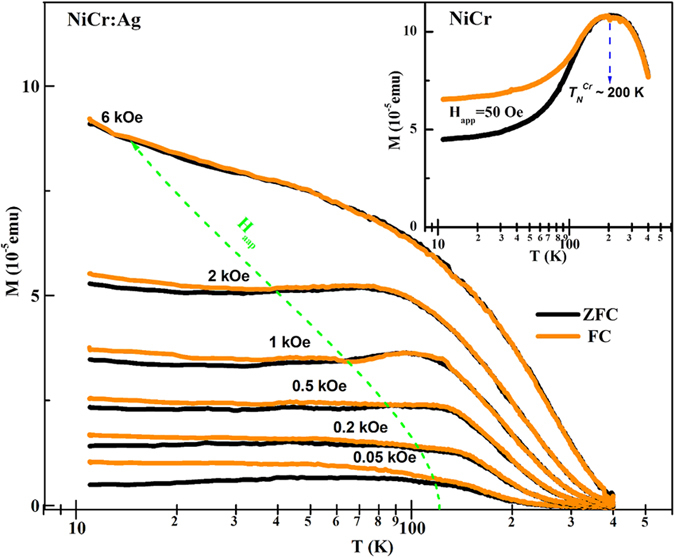
*ZFC* and *FC* magnetization curves of Ni_95_Cr_5_:Ag nanocomposite as a function of temperature (log scale). Measuring applied field *H*_*app*_: 0.05 kOe; 0.2 kOe; 0.5 kOe; 1 kOe; 2 kOe; 6 kOe. Inset shows ZFC and FC magnetization curves measured at *H*_*app*_ = 0.05 kOe for bare Ni_95_Cr_5_ nanoalloy film.

To this end, the temperature dependence of magnetizations of these Ni_95_Cr_5_:Ag nanocomposites in *ZFC* (zero-field-cooled) and *FC* (field-cooled) conditions were recorded at various applied magnetic fields, *H*_*app*_, from 0.05 to 6 kOe, as shown in Fig. 1. Various features of interest are present in this figure: first, the *ZFC* and *FC* curves almost coincide at high temperatures, but diverge markedly with decreasing temperatures. This constitutes first clear evidence for the presence of magnetic Ni_95_Cr_5_ nanograins immersed in the non-magnetic Ag matrix. Moreover, a broad peak can be seen ending around the temperature range of 100−150 K in *ZFC* curve at low fields (*H*_*app*_ = 0.05 kOe), which resembles the spin blocking temperature, *T*_*B*_, above which Ni_95_Cr_5_ nanograins should show superparamagnetic (SPM) behavior. The broad nature of the *T*_*B*_peak emanates from the non-negligible size distribution of the Ni_95_Cr_5_ nanograins. As *H*_*app*_ increases, the *T*_*B*_ peak moves towards low temperatures (as indicated by the green dotted line), eventually vanishing at *H*_*app*_ of 6 kOe, where *ZFC* and *FC* curves almost fully coincide with each other. Finally, as shown in Inset Fig.1, bare Ni_95_Cr_5_ films, without the presence of Ag, show an antiferromagnetic transition at temperature, *T*_*N*_, around 200 K. This is a typical feature of Cr-segregation^3^, below which both *FC* and *ZFC* magnetizations decrease. Interestingly, the *FC* curve of the Ni_95_Cr_5_:Ag nanocomposite shows almost the temperature independent behavior below *T*_*B*_; thus, Cr-segregation was effectively prevented due to protection by the Ag matrix. The field-dependent *ZFC* peak (*T*_*B*_) and near-constant *FC* magnetization curves below *T*_*B*_ indicate spin-glass type features^16^,^17^,^18^, possibly due to a slight surface-spin disorder caused by interfacial interaction with the Ag matrix. Thus, Ni_95_Cr_5_ nanograins behave more like a core-shell spin-structure with an ordered, ferromagnetic Ni_95_Cr_5_ core, represented by *T*_*B*_, surrounded by a spin-disordered shell.

**Figure 2:**
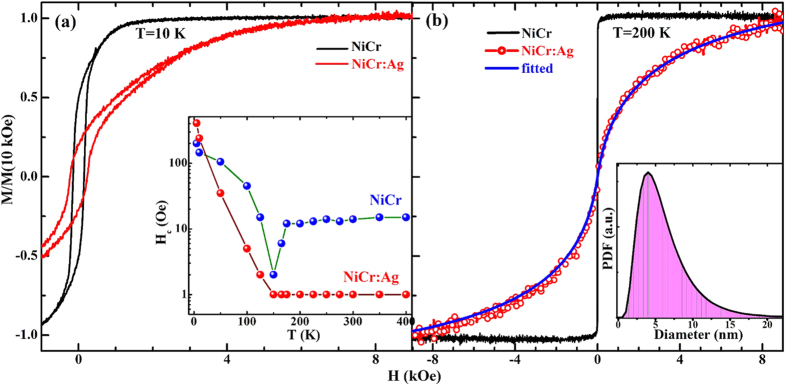
Magnetic hysteresis behavior below and above blocking temperature of Ni_95_Cr_5_:Ag nanocomposite. Normalized *M*−*H* loops for Ni_95_Cr_5_:Ag nanocomposite and Ni_95_Cr_5_ nanoalloy films were taken at (a) 10 K and (b) 200 K. The inset of Fig. 2(a) shows *H*_*c*_ vs. *T* curves for both Ni_95_Cr_5_:Ag and Ni_95_Cr_5_ films. The diameter distribution (probability density function) (inset of Fig. 2(b)) extracted from a Langevin fit [Eq. (1)] to 200 K *M−H* loops (blue color) of Ni_95_Cr_5_:Ag.

For a better understanding of the observed SPM behavior, magnetization hysteresis (i.e. *M*−*H* loops) was measured as a function of applied magnetic field up to 10 kOe under *ZFC* conditions. Such representative normalized *M*−*H* loops, taken at 10 K for both Ni_95_Cr_5_:Ag and Ni_95_Cr_5_ films, are shown in Fig. 2(a). The *M*−*H* loop of the Ni_95_Cr_5_:Ag nanocomposite (red curve) does not saturate easily under applied field of up to 8 kOe, compared to the low field (≤2 kOe) required for the magnetically soft Ni_95_Cr_5_ nanoalloy film (black curve). The coercivity value (*H*_*c*_) of the Ni_95_Cr_5_:Ag nanocomposite (~265 Oe) is almost twice as large as that of the Ni_95_Cr_5_ nanoalloy (~135 Oe). There could be many possible reasons for this observation, such as enhancement in surface anisotropy due to the interfacial roughness of Ag/Ni_95_Cr_5_ (discussed in below) causing a more canted-type Ni_95_Cr_5_ core spin structure; alternatively, interface contact of the Ni_95_Cr_5_-nanoalloy with the Ag matrix can result in some hybridization of Ag and Ni_95_Cr_5_ orbitals, as has been explained elsewhere^10^,^11^. Strikingly, *H*_*c*_ values of the Ni_95_Cr_5_:Ag nanocomposite, deduced from *M*−*H* loops measured between 5−400 K, almost fall to zero above the blocking temperature, *T*_*B*_ = 150 K, clearly indicating SPM Ni_95_Cr_5_ nanograins in the Ag matrix (Inset of Fig. 2(a)). Around the same temperature (*T*_*B*_), the magnetoresistance value (shown in supplementary information Fig. S2) of Ni_95_Cr_5_:Ag nanocomposite abruptly decreases from 0.9% to 0%, clearly verifying the SPM behavior of Ni_95_Cr_5_ nanograins in Ag matrix.

Another meaningful conclusion can be drawn from the temperature dependence of *H*_*c*_ values of the Ni_95_Cr_5_ nanoalloy film (Inset of Fig. 2(a)) which show sharp minima around the antiferromagnetic transition temperature (*T*_*N*_) 150 K of Cr. The low value of *T*_*N*_compared to the bulk Cr value (315 K)^5^,^7^,^9^, can be attributed to the nano sizes of Cr-segregates. These samples also display step-type *M-H* loops (green curve) with a constant *H*_*c*_ value (15 Oe) above *T*_*N*_, which is an expected feature of NiCr nanoalloys, but the increase of the *H*_*c*_ value below *T*_*N*_ can be attributed to the uncompensated magnetization of NiCr cores over Cr-segregates. We did not observe such features in Ni_95_Cr_5_:Ag nanocomposite (brown curve), which, once more, supports our argument that the Ni_95_Cr_5_:Ag nanocomposite does not show any Cr-segregation. In the present case, the *T*_*N*_ of the Ni_95_Cr_5_ nanoalloy films (150 K) and the *T*_*B*_of the Ni_95_Cr_5_:Ag nanocomposite (120 K) are found to be close, but one should not confuse them, since the Ni_95_Cr_5_:Ag nanocomposite shows zero *H*_*c*_ values above *T*_*B*_ due to SPM behavior, as opposed to the non-zero constant value in case of the Ni_95_Cr_5_ nanoalloy film.

The exact size range of the core NiCr nanograins was investigated next. SPM Ni_95_Cr_5_ nanograins show typical *S*-shape *M−H* loop above *T*_*B*_ (200 K, presented in Fig. 2(b)), with zero *H*_*c*_ and remnant magnetization values. The *M*−*H* loops of ideal SPM non-interacting nanoparticles of various sizes can be fitted by a log-normal moment-weighted Langevin function^19^:


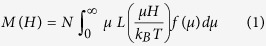


where 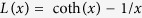,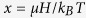 is the Langevin function and


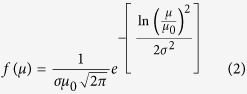


is the log-normal distribution, where *μ*_0_ is the median moment and *σ* is the standard deviation of ln(*μ/μ*_o_), respectively. These distribution parameters, *μ*_*o*_ and *σ*, are estimated by fitting Eq. (1) to the *M*−*H* data of 200 K (*i.e.* above *T*_*B*_). From our best fit shown in Fig. 2(b) (blue line), we deduced a log-normal distribution (Inset of Fig. 2(b)) of core diameters with mean diameter value *D*_o_ = 5.32 ± 0.542 nm.

**Figure 3:**
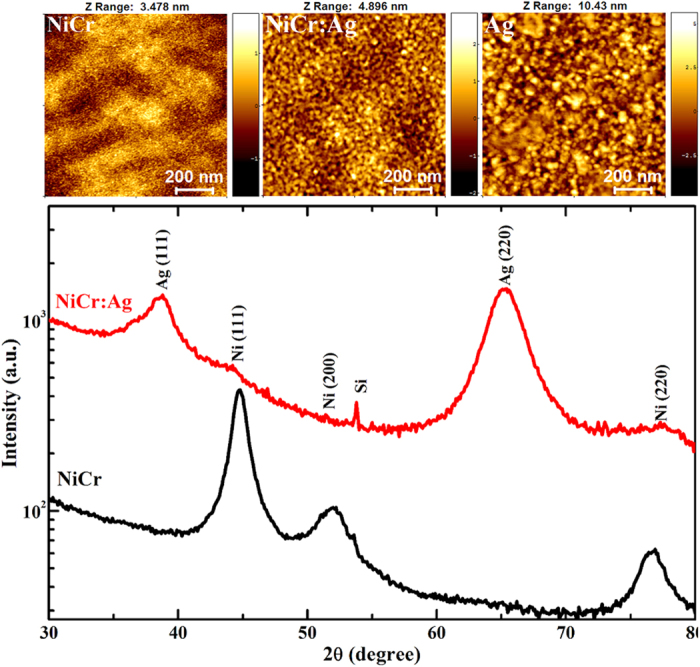
Crystalline structure and surface morphology of Ni_95_Cr_5_:Ag nanocomposite and Ni_95_Cr_5_ film. GIXRD patterns of Ni_95_Cr_5_:Ag nanocomposite and Ni_95_Cr_5_ nanoalloy film. AFM images of Ni_95_Cr_5_, Ni_95_Cr_5_:Ag and Ag films (over 1 μm × 1 μm area) are shown in upper panel. The measured RMS roughness values are 0.380, 0.532, and 1.319 nm for the as deposited Ni_95_Cr_5_, Ni_95_Cr_5_:Ag and Ag film, respectively.

Because of the small sizes of Ni_95_Cr_5_ grains in the Ag matrix, the GIXRD (lower panel of Fig. 3) pattern shows only faint ‘shoulders’ around the position of FCC-Ni (111) and (220) peaks compared to the Ni_95_Cr_5_ nanoalloy. The corresponding surface morphology changes can be seen in AFM images (upper panel of Fig. 3), with a sharp reduction in surface RMS roughness in Ni_95_Cr_5_:Ag nanocomposite film (~0.532 nm) compared to pure Ag film (~1.319 nm). Surface roughness is highly dependent on deposition rate, grain size and thickness of films^11^. The Ni_95_Cr_5_ film shows reduced roughness due to lower deposition rate and smaller grain size compared to the Ag film. The roughness changes in the Ni_95_Cr_5_:Ag case, where there are altogether different grain sizes. The GIXRD pattern does not show any impurity antiferromagnetic phases such as Cr, CrO_2_, Cr_2_O_3_ or NiO, which is further confirmed by exchange bias study (shown in supplementary information Fig. S3) at 3 K, a method described elsewhere^11^,^20^.

**Figure 4:**
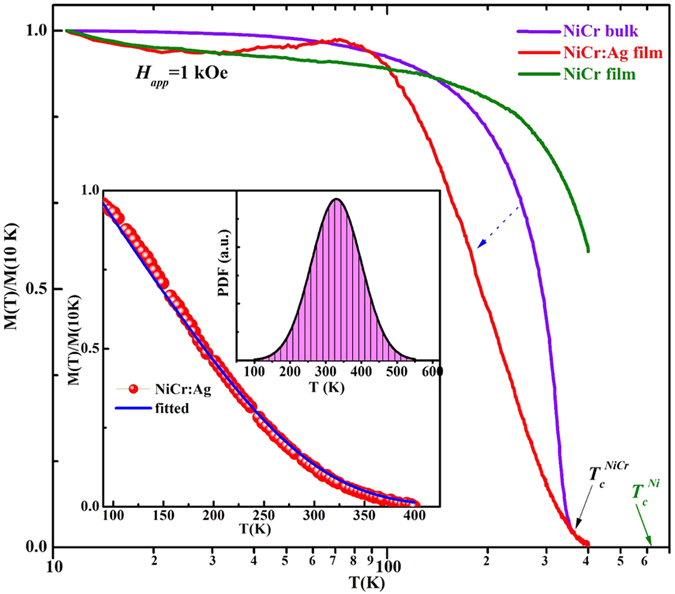
Curie temperature (*T*_*C*_) determination of Ni_95_Cr_5_:Ag nanocomposite and Ni_95_Cr_5_ film. Normalized magnetizations at fixed field, *H*_*app*_ = 1 kOe, as a function of temperature, *T*, (log scale) for Ni_95_Cr_5_:Ag nanocomposite, and Ni_95_Cr_5_ nanoalloy films along with bulk Ni_95_Cr_5_ alloy. The inset shows the fitted data to Eq. (4) of Ni_95_Cr_5_:Ag nanocomposite in the critical range 90 −400 K, which results in *T*_*C*_ distribution (probability density function).

Prevention of Cr-segregation is, of course, just a means to an end; the main goal is to preserve the desired magnetic properties of bulk NiCr in the nanoscale, for targeted applications. To investigate whether this goal was achieved, normalized *FC* magnetization *M* (*T*)/*M* (10 K) measured under an applied field of *H*_*app*_ = 1 kOe in the temperature range 5 K ≤ *T* ≤ 400 K for Ni_95_Cr_5_:Ag nanocomposite and Ni_95_Cr_5_ nanoalloy are shown in Fig. 4. The *M-T* data of bulk Ni_95_Cr_5_ alloy is also given in the same figure for comparison. The ratio *M*/*H* closely approximates the initial differential susceptibility *χ = dM*/*dH* so that a rough *T*_*C*_estimation can be achieved without substantial field-induced broadening. The *M* (*T*)/*M* (10 K) vs. temperature curve of Ni_95_Cr_5_:Ag nanocomposite shows qualitatively different behavior from that of the ordered bulk Ni_95_Cr_5_ alloy, with its magnetization decreasing earlier, in the temperature range 100−400 K; however, it displays the same *T*_*C*_ as the bulk sample.

Let us scrutinize this observation a bit further. Amekura *et al.*^21^ observed similar behavior in SPM Ni nanoparticles (~3 nm) embedded in SiO_2_ matrix; however, a small but non-zero value of magnetization remained in the nanoparticles even above the bulk *T*_*C*_. They ascribed this to the finite size effects of nanoparticles using quantum Monte-Carlo simulation. Skomski *et al.*^22^ also predicted similar results for the *T*_*C*_of interaction-free multiphase nanostructures theoretically, and argued that in nanocomposites there can only be one *T*_*C*_ regardless of the bulk Curie temperatures of the phases involved. The *T*_*C*_is higher than the volume average of the Curie temperatures of the individual phases. Skomski *et al.*^22^ also explained that because of the non-relativistic character of the interatomic exchange, the *T*_*C*_ coupling range is atomic rather than nanoscale, so that the comparatively high Curie temperatures of nanocomposites do not translate into an enhanced permanent-magnet energy product (*B−H*), i.e. hysteresis. In a similar fashion, our Ni_95_Cr_5_:Ag nanocomposite contains a great (approximately infinite) number of SPM clusters, which do not interact with each other above *T*_*B*_, due to the presence of the Ag matrix. Each cluster is characterized by a specific *T*_*C*_ value, and its magnetization near this *T*_*C*_ follows the general power law^23^ given by:


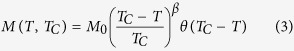


where *M*_*o*_ is a factor proportional to the saturation magnetization, and *θ* is the Heaviside function, pinning *M* to zero for temperatures above *T*_*C*_, thus warranting that the material has entered its paramagnetic phase^23^. The overall magnetization of the model thin film can be derived by^23^:


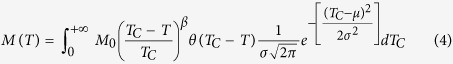


where *σ* and *μ* is the *T*_*C*_ variance and mean of distribution, respectively. We fitted the parameters of this function to the experimental magnetization graph at elevated temperatures, near *T*_*C*_ using the least squares method, assuming that the mean of distribution (*μ*) is the *T*_*C*_obtained experimentally (330 K). Our best fit is shown in Inset of Fig. 4, along with the probability distribution function (PDF) plot, for *σ* = 70 K and *β* = 1.12, showing excellent agreement with the experiment. Some part of the *T*_*C*_ distribution (around ~5−12 K) can be attributed to applied field induced broadening;^23^ however, the remaining *T*_*C*_ distribution is ascribed to the intrinsic contribution of different sizes of Ni_95_Cr_5_ nanograins in the Ag matrix. Thus, our Ni_95_Cr_5_:Ag nanocomposite indeed exhibit a *T*_*C*_ distribution around the bulk Ni_95_Cr_5_ alloy *T*_*C*_ value under control conditions.

To demonstrate the effectiveness of our method in protecting Cr-segregation, it is imperative to emphasize that the magnetization of the bare Ni_95_Cr_5_ nanoalloy (green curve, Fig. 4) does not vanish at bulk Ni_95_Cr_5_ *T*_*C*_ value of around 320 K, clearly suffering from Cr-segregation, with the resultant deterioration of its magnetic behavior. Even though the thermal demagnetization process of our Ni_95_Cr_5_:Ag nanocomposite is somewhat different than of the bulk Ni_95_Cr_5_, the complete loss of magnetization happens at around the same temperature as with bulk Ni_95_Cr_5_ alloy, which rules out any possibility of Cr-segregation in our Ni_95_Cr_5_:Ag nanocomposite.

## Discussion

**Figure 5:**
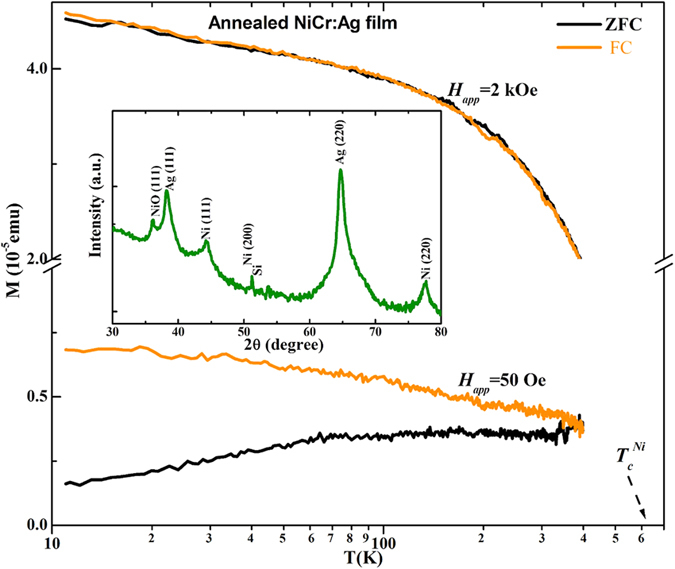
Structural and magnetic property change after post annealing. *ZFC* and *FC* magnetizations as function of temperature, *T*, (log scale) measured at a fixed field of 50 Oe and 2 kOe for annealed Ni_95_Cr_5_:Ag nanocomposite. The inset shows GIXRD pattern of annealed Ni_95_Cr_5_:Ag nanocomposite.

After protecting Ni_95_Cr_5_ nanoalloys in Ag matrix from suffering Cr-surface segregation, it is informative to test the temperature limitations of our methods by comparing the magnetic properties of the Ni_95_Cr_5_:Ag nanocomposite before and after annealing. The temperature dependence of *ZFC* and *FC* magnetizations curve measured at low (50 Oe) and high field (2 kOe) for Ni_95_Cr_5_:Ag nanocomposites annealed at 450 ^o^C under vacuum of 1 × 10^−7^ mbar are plotted in Fig. 5. A large difference between *ZFC* and *FC* magnetizations (at 50 Oe) starting well above room temperature can be observed, indicating a higher *T*_*B*_ ≥ 400 K, whereas for the same field as-deposited Ni_95_Cr_5_:Ag nanocomposite show low *T*_*B*_ around 100 K. This difference clearly indicates an enlargement of effective magnetic volume due to either increased Ni_95_Cr_5_ particle sizes or Cr-segregation in the Ag non-magnetic matrix upon annealing.

This is further supported by the GIXRD result of the Inset of Fig. 5, showing much sharper Ni peaks compared to those of the as-deposited film (Fig.3, red curve). FCC Ni (111), (200) and (220) diffraction peaks obviously appear, signifying the formation and precipitation of FCC Ni_95_Cr_5_-rich particles of sizes ~10−12 nm (estimated by the Scherrer formula^24^) from the Ag-matrix. It is worth mentioning here that the addition of such a small percentage of Cr does not offset the XRD peak positions of Ni noticeably, because it hardly induces any significant change in the Ni lattice^25^. The NiO (111) peak can only be observed due to surface oxidation of Ni_95_Cr_5_ nanoparticles of a relatively larger size, as they emerge from the Ag matrix after annealing and get exposed to air during transfer for the GIXRD measurement. These results are in agreement with observations reported on Ni-Ag, Co:Ag and Co:Cu nanogranular systems^25^−^28^. In the GIXRD of the as-deposited Ni_95_Cr_5_:Ag nanocomposite, Ni_95_Cr_5_-rich particles cannot be seen clearly, due to the formation of very small clusters and the metastable alloying with Ag. Although it is known that at equilibrium conditions the mutual solubility of Ni_95_Cr_5_ and Ag is very low, the use of a non-equilibrium deposition process such as sputtering at room temperature allowed a substantial concentration of Ni_95_Cr_5_ clusters to dissolve, forming Ni_95_Cr_5_:Ag nanocomposites;^26^−^29^ their concentration, however, was reduced after annealing, through an extensive demixing process.

At high field (2 kOe), *ZFC* and *FC* magnetizations overlap and do not vanish at bulk Ni_95_Cr_5_ alloy *T*_*C*_value of 320 K, but instead approach towards Ni bulk *T*_*C*_ values similar to the Ni_95_Cr_5_ nanoalloy films (Fig. 4, green curve), showing that some Cr-segregation is also caused by annealing. Similar behavior was observed previously in pure Ni_95_Cr_5_ nanoclusters after annealing^6^. Therefore, there are two simultaneous segregation mechanisms at play: Ni_95_Cr_5_ nanograins segregating from the Ag matrix, due to dewetting, and precipitating into larger Ni_95_Cr_5_ grains also allow for Cr-surface segregation in each one of these grains. Their combination eventually enhances Ni−Ni particle interactions;^6^,^9^ as a result, bulk-Ni magnetic properties (higher *T*_*B*_ and *T*_*C*_ values) are expected. It should be stressed, however, that annealing took place at distinctly higher temperatures than those required for potential applications of our nanocomposite, and, unlike previously reported case-studies, imposes no practical limitation in the utilization of our method.

In summary, this study tackles a frequent problem of bimetallic M-Cr nanoalloys: that of Cr-surface segregation, with the resultant deterioration of magnetic properties. Ni_95_Cr_5_ nanoalloys were synthesized, immersed in a non-magnetic Ag matrix via a direct co-sputtering technique. The strong correlation between structure and magnetic properties enabled the collection of information about the structure of the nanoalloys by performing magnetic measurements. Low-temperature divergence of *ZFC* and *FC* magnetizations certified the presence of Ni_95_Cr_5_ nanoalloys of a non-negligible size distribution. The observed SPM behavior of the nanocomposite was investigated by measuring magnetization hysteresis; indeed, the *H*_*c*_ value dropping to zero above the *T*_*B*_ not only re-confirmed the presence of SPM Ni_95_Cr_5_ nanoalloys in the Ag matrix, but also revealed their size distribution. Most importantly, averting Cr from demixing preserves the desired magnetic properties of bulk NiCr (*e.g*. *T*_*C*_) in the nanoscale for targeted applications, such as magnetic hyperthermia for cancer treatment. Once more, the necessity for the segregation prevention method was confirmed by demonstrating that without the presence of the Ag matrix Ni_95_Cr_5_ nanoalloys unequivocally suffer from Cr-segregation. Finally, temperature limitations for the usage of our method were tested by comparing magnetic properties of the Ni_95_Cr_5_:Ag nanocomposite before and after annealing.

## Methods

### Nanocomposite growth

The Ni_95_Cr_5_:Ag nanocomposite films (~80 nm thick) were fabricated by a co-sputtering technique, and deposited on Si and fused quartz substrates at ambient temperature. Schematic diagram of experimental setup is shown in Fig. S4. The composition was adjusted by optimizing the DC magnetron sputtering power of the Ni_95_Cr_5_ (40 W) and Ag (20 W) targets while maintaining Ar pressure at 2.7 × 10^−3^ mbar. The deposit thickness rate was measured by using a quartz-crystal monitor. We deliberately chose a deposition rate with a low Ni_95_Cr_5_ (0.04 nm/s) volume fraction, so that the Ni_95_Cr_5_ particles remained isolated in the Ag medium. To ensure that they were fully capped by a layer of Ag, we prolonged the deposition of Ag (0.09 nm/s). Substrate table rotation was set at 2 rpm for all depositions, to ensure uniform film deposition. To investigate the effect of post-thermal treatment, the nanocomposites were subsequently annealed under vacuum lower than 1 × 10^−7^ mbar for 60 minute at 450 ^o^C in the deposition chamber. Nanoalloy Ni_95_Cr_5_ thin films were also deposited under the same sputtering power (40 W) for comparative study.

### Surface morphology and Compositional analysis

After substrate landing, nanocomposite-loaded Si (100) substrates were load-lock transferred to an inert gas (N_2_) glove-box and surface morphology was characterized by atomic force microscopy (AFM) using a Multimode 8 (Bruker, Santa Barbara, CA) instrument operating in tapping mode. The AFM system height “Z” resolution and noise floor are less than 0.030 nm. The scanning probe processor (SPIP) (Image Metrology, Hørsholm, DK) software was employed for the root-mean-square (RMS) roughness analysis. Nanocomposite structures were characterized using both grazing incidence x-ray diffraction (GIXRD, Bruker D8 Discover XRD^2^ system with Cu *K*_α_ x-ray source) at grazing angle of 0.25^o^ and Cs-corrected transmission electron microscopy (TEM, FEI Titan G2^TM^ 80−300 kV) operating at 300 kV. The annular bright-field (ABF) image was taken on a scanning transmission electron microscope. Energy dispersive x-ray spectroscopy was performed with an Oxford Xmax system, with an 80 mm^2^ silicon drift detector (SDD) and energy resolution of 136 eV.

### Magnetic measurements

The magnetic properties of the as-deposited and annealed films were measured with in-plane configuration in a Quantum Design physical property measurement system (PPMS^TM^) using vibrating sample magnetometer (VSM, 2−400 K). Magnetization as a function of applied magnetic field, *M*−*H*, loops were taken at various temperatures between 5 and 400 K. The diamagnetic contribution from the Si substrate, glue and Ag was subtracted from magnetization data by measuring the high-field magnetic susceptibility. For zero-field-cooled (*ZFC*) magnetization measurements, the sample was initially cooled to 5 K in zero field, and subsequently magnetizations were measured under various fixed fields upon heating. Next, the field-cooled (*FC*) magnetization was recorded during cooling for each field.

## Additional Information

**How to cite this article**: Bohra, M. *et al.* Control of Surface Segregation in Bimetallic NiCr Nanoalloys Immersed in Ag Matrix. *Sci. Rep.* **6**, 19153; doi: 10.1038/srep19153 (2016).

## Supplementary Material

Supplementary Information
