# Supplementary material for: Control of Surface Segregation in Bimetallic NiCr Nanoalloys Immersed in Ag Matrix
Source: Sci Rep. 2016 Jan 11;6:19153. doi: 10.1038/srep19153 (PMC4707502; doi:10.1038/srep19153)
Supplement: Supplementary Information [file srep19153-s1.pdf]

Supplementary Information

Control of Surface Segregation in Bimetallic NiCr Nanoalloys Immersed in Ag Matrix

Murtaza Bohra,[1,2*] Vidyadhar Singh,[1] Panagiotis Grammatikopoulos,[1] Evropi Toulkeridou,[1] Rosa E. Diaz,[1] Jean-François Bobo,[3] and Mukhles Sowwan[1,4*]

[1]Nanoparticles by Design Unit, Okinawa Institute of Science and Technology Graduate University, 1919-1 Tancha Onna-Son, Okinawa, 904-0495, Japan.

[2]Mahindra Ecole Centrale, Survey no: 62/1A, Bahadurpally Jeedimetla, Hyderabad-500043, Telangana India

[3]Centre d'Elaboration de Materiaux et d'Etudes Structurales (CEMES), 29 rue Jeanne Marvig, 31055 Toulouse Cedex 4, France.

[4]Nanotechnology Research Laboratory, Al-Quds University, East Jerusalem, P.O. Box 51000, Palestine.

*Corresponding author: mukhles@oist.jp (M.S.); murtaza@gmail.com (M.B.)

Supplementary Figures:

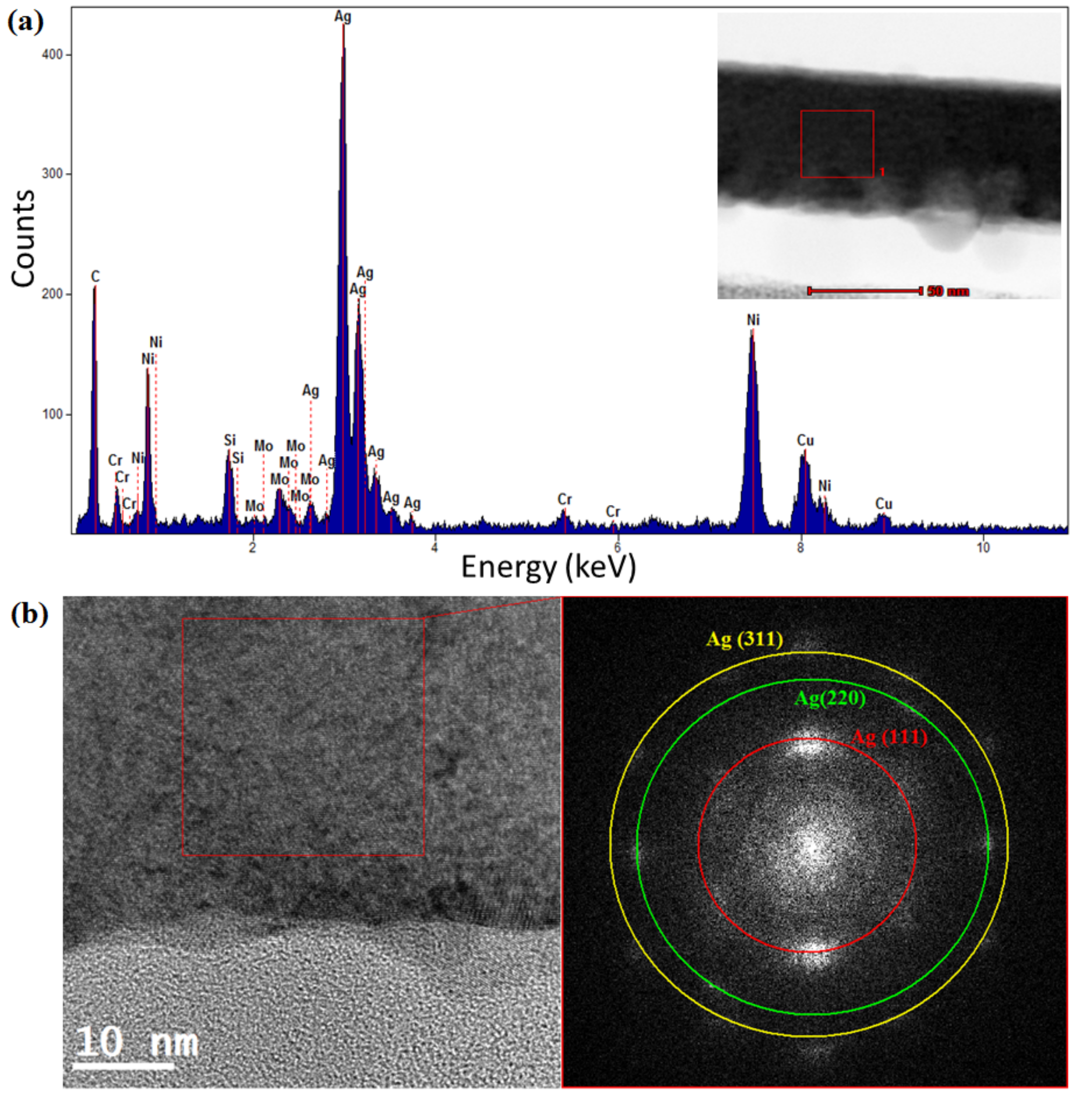

Fig. S1 Element-specific analysis and cross section HRTEM images of $Ni_{95}Cr_5$:Ag nanocomposite. **(a)** The energy-dispersive X-ray spectroscopy spectrum of $Ni_{95}Cr_5$:Ag nanocomposite on Si substrates with the annular bright-field STEM image (inset). The C, Cu, Mo and Si peaks are attributed to the background of TEM lamella and substrates. The relative atomic concentrations are estimated around to be $Ni_{95}Cr_5$ (~35%): Ag (~65%) **(b)** Cross section HRTEM image of Ni95Cr5:Ag nanocomposite with a Fourier transform analysis shows only dominate Ag phase because of thoroughly intermixing of $Ni_{95}Cr_5$ grains into Ag matrix.

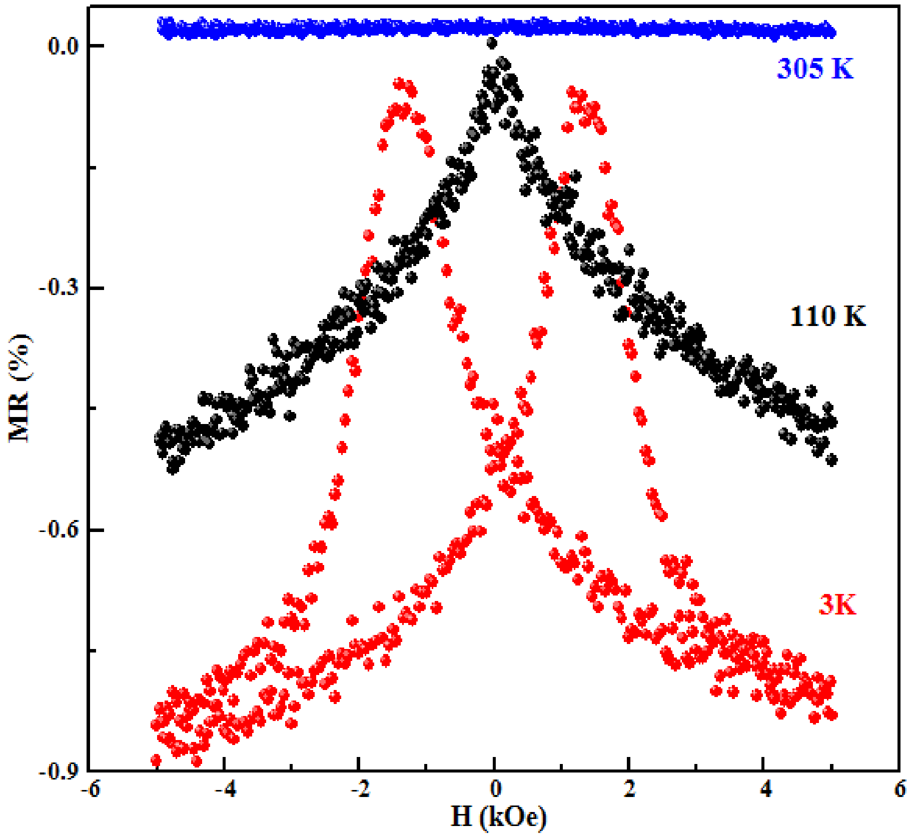

Fig. S2 Magnetoresistance (MR) curves for $Ni_{95}Cr_5$:Ag nanocomposites at different temperatures 3, 110 and 305 K. The MR decreases sharply from 0.9% to 0% when temperature increases from 3 K to 305 K. Such an abrupt decrease in MR above T_B clearly specifies SPM behavior. Below T_B, when the spin of ferromagnetic $Ni_{95}Cr_5$ nanograins is frozen in the Ag matrix, one can observe a high MR value for coercivity of 1.5 kOe at 3 K. This coercivity is three times higher than the one measured at 3 K in *M–H* loops, inferring that other factors also affect spin scattering.

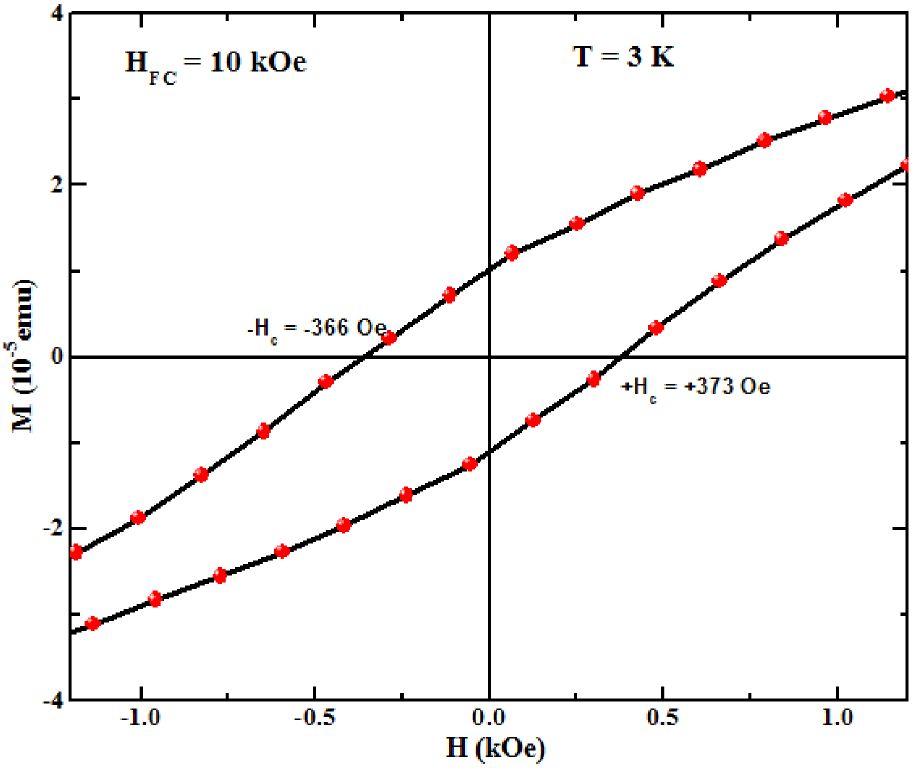

Fig. S3 Magnetic exchange bias study for surface oxidation examination. *M*–*H* loops taken at 3 K after field cooling of sample from 400 K under field of 10 kOe. No any signature of loop shift and coercivity enhancement is observed within accuracy limit, indicating absence of exchange bias in $Ni_{95}Cr_5$:Ag nanocomposites. This result rules out any possibility of Cr-segregation and thin oxide shell (Cr_2O_3 and NiO) formation.

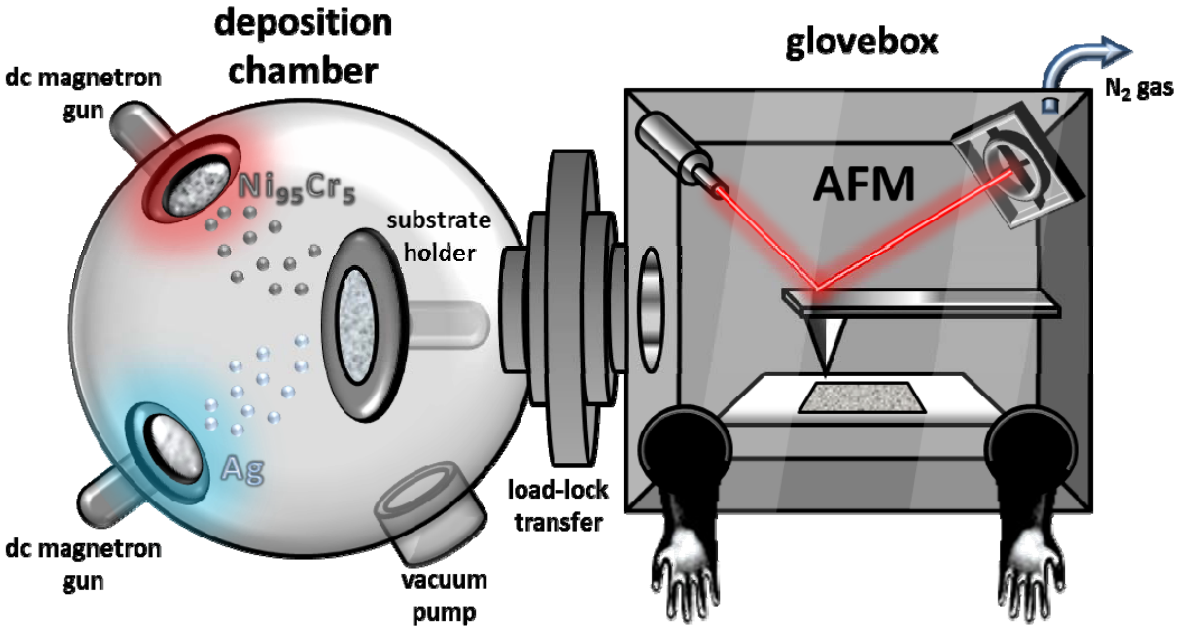

Fig. S4 Schematics of experimental setup for co-sputtering. DC magnetron sputtering system comprising two sputter targets (NiCr and Ag) for co-deposition, accompanied by load lock system equipped with the atomic force microscope.
